# Soft tissue invasion of papillary thyroid carcinoma

**DOI:** 10.1007/s10585-016-9800-3

**Published:** 2016-05-06

**Authors:** Jen-Der Lin, Chuen Hsueh, Tzu-Chieh Chao

**Affiliations:** Division of Endocrinology and Metabolism, Department of Internal Medicine, Chang Gung Memorial Hospital, Chang Gung University, 5, Fu-Shin St., Kweishan County, Taoyuan Hsien Taiwan, ROC; Department of Pathology, Chang Gung Memorial Hospital, Chang Gung University, Kweishan County, Taiwan, ROC; Department of General Surgery, Chang Gung Memorial Hospital, Chang Gung University, Kweishan County, Taiwan, ROC

**Keywords:** Total thyroidectomy, Thyroglobulin, Radioactive iodide, Cancer-specific mortality

## Abstract

Extrathyroidal extension (ETE) of papillary thyroid carcinoma (PTC) is common and clinical presentation can vary from minimal to extensive locoregional involvement. Although PTC is generally considered the most benign among all thyroid carcinomas, it may present with local invasion with poor prognosis. Our retrospective study involved 3267 PTC patients undergoing regular follow-up at Chang Gung Medical Center in Linkou, Taiwan. Among them, 269 were PTC cases with ETE, having tumors greater than 1 cm in size and treated with total or complete thyroidectomy with or without lymph node dissection for which the follow-up period was over 10 years. The mean age of 269 cases was 46.8 ± 15.1 (range 11–83 years) years. The number of females was 204 (75.8 %). Patients were categorized into minimal ETE (175 cases) and extensive ETE (94 cases) groups according to surgical findings and pathological reports. Mean follow-up period was 13.3 ± 5.5 (range 0.2–29.3) years, during which 28 (10.4 %) patients died of thyroid cancer; and 63 (23.4 %) of all-cause mortality. Multivariate analysis showed that age, gender, extensive ETE, and lymph node metastasis had a statistically significant effect on thyroid cancer mortality. Survival rates were significantly different between minimal ETE and extensive ETE groups (*p* < 0.0001). In conclusion, perithyroidal soft tissue involvement by PTC is an important factor that determines patient prognosis and a closer follow-up and more aggressive treatment is necessary for patients who are old, male, extensive ETE, and with lymph node involvement.

## Introduction

Locoregional extension to lymph nodes and perithyroidal soft tissues is the main characteristic of papillary thyroid carcinoma (PTC) [1, 2]. Extrathyroidal invasion by PTC may be classified as stage T3 with minimal extrathyroidal extension (ETE), whereas PTC extending beyond the thyroid capsule to invade subcutaneous soft tissue, larynx, trachea, esophagus, or recurrent laryngeal nerve as stage T4, under the TNM staging system [3]. Complete surgical removal of PTC with locoregional extension may have a good prognosis [4]. However, a positive surgical margin is not unusual after thyroidectomy, especially in patients with stage T4 cancer. Administration of postoperative adjuvant therapies and long-term follow-up are mandatory for these cases [5, 6]. We currently have insufficient information regarding the long-term follow-up and prognosis associated with minimal (T3) and extensive (T4) ETE of PTC patients. A retrospective review of a prospective database established by the Endocrinology Division in 1995 was performed in order to assess the long-term therapeutic outcome and prognostic factors associated with PTC with ETE. The PTC with ETE patients who had a minimum follow-up period of 10 years were identified. Locoregional recurrence or distant metastases were analyzed in these patients following radioactive iodine (^131^I) treatment and other adjuvant therapies.

## Subjects and methods

**Fig. 1:**
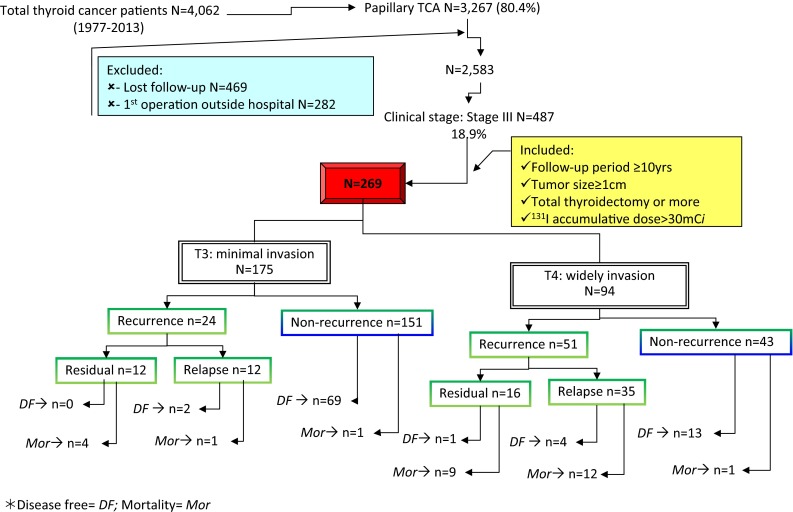
Papillary thyroid cancer patients with soft tissue invasion and long-term follow-up were selected from 4062 thyroid cancer patients. Two hundred and sixty-nine cases were categorized in minimal invasion and extensive invasion according to Union for International Cancer Control tumor-node-metastasis (TNM) criteria (6th edition)

During the period 1977 and 2013, a total of 2583 PTC from 4062 thyroid cancer patients underwent thyroidectomy and regular follow-up care at Chang Gung Medical Center in Linkou, Taiwan (Fig. 1). Among them, 487 patients with soft tissue (ST) invasion, 269 of which had tumors greater than 1 cm in size and received total or near-total thyroidectomy with or without lymph node dissection and underwent follow-up for over 10 years. In addition, there were 599 intra-thyroid PTC, 127 with lymph node metastasis, and 51 with distant metastasis met the inclusion criteria.

The mean age of 269 selected patients who had PTC with ST invasion, where 204 (75.8 %) were females, was 46.8 ± 15.1 (range 11–83 years) years. Patients were classified as stage T3 (minimal ETE) or T4 (extensive ETE) according to operative findings and pathological reports. Among them, 175 (65.1 %) patients had minimal ETE. Of these 269 patients, 132 patients (49.1 %) underwent lymph-node dissection. There were 49 (18.2 %) cases treated with locoregional neck re-operation for the local recurrent PTC. All patients were staged using the Union for International Cancer Control tumor-node-metastasis (TNM) criteria (6th edition) [3]. All thyroid carcinoma tissues were pathologically classified according to the World Health Organization criteria [7]. There were 10 follicular variants of PTC, 4 cases with a poorly differentiated compartment, and 1 tall-cell variant. There were 65 cases (24.2 %) with multifocal PTC.

In our center, PTC patients with histologically verified locoregional or distant metastases were recommended to undergo thyroid radioiodine remnant ablation at 4–6 weeks after thyroidectomy. The dose of ^131^I ablation for most patients was 30–100 mCi (1.1–3.7 GBq). One week after ^131^I administration, whole-body scan (WBS) was performed using a dual-head gamma camera (Siemens Medical Solutions USA, Inc., USA) equipped with a high-energy collimator. The whole-body image was acquired using continuous mode scanning at a speed of 5 cm/min. In addition, thyroid scintigraphy was performed using a pinhole collimator with a 4-mm aperture placed 7 cm above the neck for a total of 50,000 counts or 30 min. Levothyroxine treatment was initiated to decrease thyroid stimulating hormone (TSH) levels without inducing clinical thyrotoxicosis. Cases where ^131^I uptake extended beyond the thyroid bed were classified as persistent disease or metastasis unless proven to be a false positive. Higher therapeutic doses of 3.7–7.4 GBq (100–200 mCi) were administered to these patients. Patients receiving doses exceeding 1.1 GBq were isolated at hospital admission. WBS was performed 2 weeks after the administration of the higher therapeutic dose of ^131^I. According to the radiation regulations in Taiwan, patients receiving <1.1 GBq are classified as outpatients. Radioactive avidity of the lesions was determined in the first positive WBS. External radiotherapy was performed for symptomatic relief of bone metastasis.

In patients without detectable ^131^I uptake beyond the thyroid bed during the post-ablation WBS, thyroid hormone treatment was withdrawn after 6–12 months, and thyroglobulin (Tg), TSH, and anti-Tg antibodies were measured. Serum Tg levels was measured using an immunoradiometric assay kit (CIS Bio International, Gif Sur Yvette, France). The Tg level was considered accurate only if the recovery test (performed in all serum samples) was less than 80 %. The following data were collected from admission records: age, gender, primary tumor size, ultrasonographic findings, results of fine needle aspiration cytology, preoperative thyroid function, surgical methods, histopathological findings, TNM staging, 1-month postoperative serum Tg levels, presence of Tg antibodies, diagnostic and therapeutic ^131^I WBS results, ^131^I avidity of the first distant metastasis, ^131^I accumulated dose, postoperative chest X-ray findings, clinical status of distant metastasis determined via noninvasive radiological and nuclear medicine examinations, treatment outcomes, causes of death, and survival status.

At the end of 2013, patients were categorized into thyroid cancer mortality, non-remission, remission, and disease free groups. The remission group consisted of patients with negative ^131^I WBS results, and no evidence of local or distant metastasis upon noninvasive examination. Disease-free patient was defined as a patient in remission with undetectable Tg without levothyroxine treatment, and undetectable Tg antibodies at final follow-up. This study was approved by the Chang Gung Medical Foundation Institutional Review Board (104-3901B), and the informed consent requirement was waived because of the retrospective nature of the study.

Categorical data were compared using Chi square or Fisher’s exact test for small size data sets. Unpaired *t* tests were used to compare continuous data between groups. Cancer-related mortality was calculated and follow-up period was determined from the date of diagnosis to the date of cancer-related mortality of the last follow-up survivor. Survival rates were calculated using the Kaplan–Meier method and compared using the log-rank test [8]. A multivariate Cox proportional hazards regression model was used to estimate the mortality risk. All statistical analyses were performed using SPSS version 17.0 statistical software (SPSS Inc., Chicago, IL, USA). A *p* value <0.05 was considered statistically significant in all tests.

## Results

At the end of the mean follow-up period of 13.3 ± 5.5 (range 0.2–29.3) years, 28 (10.4 %) patients out of the total 269 died of thyroid cancer while 63 (23.4 %) died of all-cause mortality. 44 cases (16.4 %) had non-remission status. Clinical features and therapeutic outcomes associated with all patients, and those with minimal or extensive invasion are illustrated in Table 1. Male gender, larger tumor size, more advanced TNM stages, higher cancer-related and total mortality rates, and less disease-free cases were predominant in the group of patients with extensive invasion. No difference in age, postoperative serum Tg levels, multifocality, operative method, and follow-up period existed between minimal and extensive invasion groups. Twenty-nine cases (10.8 %) were diagnosed with second primary malignancy. Among them, the mortality rates in extensive ETE group having secondary primary malignancy increased to 71.4 %.

**Table 1:** Clinical features of thyroid cancer with soft tissue invasion in total, minimal extra-thyroid extension (T3), or widely extra-thyroid extension (T4) groups

Clinical characteristics	Total	Minimal ETE	Widely ETE	*P* value*
Patient number	269	175 (65.1)	94 (34.9)	
Gender (female)	204 (75.8)	144 (82.3)	60 (63.8)	0.0007
Age at diagnosis (year)	46.8 ± 15.1	45.8 ± 13.7	48.6 ± 17.2	0.1506
Mean tumor size (cm)	3.0 ± 1.5	2.6 ± 1.0	3.7 ± 1.9	0.0001
Post-operative serum Tg level after 1 month (ng/mL)	96.2 ± 515.5	56.0 ± 187.0	172.3 ± 832.3	0.0842
Multifocality	65 (24.2)	39 (22.3)	26 (27.6)	0.3262
Histology variant				0.1834
Follicular variant of papillary	10 (3.5)	7 (4.0)	3 (3.2)	
Poorly differentiation	4 (1.5)	1 (0.6)	3 (3.2)	
Tall cell	1 (0.4)	–	1 (1.1)	
Thyroid operative method				0.1171
Total thyroidectomy	137 (50.9)	83 (47.4)	54 (57.4)	
Total thyroidectomy with LN dissection	132 (49.1)	92 (52.6)	40 (42.5)	
Lymph node metastases	80 (29.7)	43 (24.6)	37 (39.4)	0.0114
Non-remission	44 (16.4)	12 (6.9)	32 (34.0)	0.0001
Follow-up period (year)	13.3 ± 5.5	13.5 ± 4.7	13.0 ± 6.8	0.4733
Post-operative ^131^I accumulative dose (mCi)	209.5 ± 253.2	157.6 ± 180.5	305.9 ± 329.4	0.0001
Cancer mortality	28 (10.4)	6 (3.4)	22 (23.4)	0.0001
Total mortality	63 (23.4)	22 (12.6)	41 (43.6)	0.0001
Disease free	89 (33.1)	71 (40.6)	18 (19.1)	0.0004
Second primary cancer	29 (10.8)	15 (8.6)	14 (14.9)	0.1109
Mortality	15 (51.7)	5 (33.3)	10 (71.4)	0.0402
Diabetes mellitus	21 (7.8)	14 (8.0)	7 (7.4)	0.8719

Number (%)

* Between Minimal ETE and Widely ETE groups

In order to assess the prognostic factors associated with thyroid cancer-related mortality, univariate analysis was performed between cancer-related mortality and survival cases, and a statistically significant difference in illustrated age, gender, extensive ETE, and tumor size was observed between two groups (Table 2). Initial surgical procedure with or without lymph node dissection did not exhibit a statistically significant difference between cancer mortality and other cases. Histological data, only lymph node metastasis showed statistical difference. Vascular invasion and multifocality were not statistically different between cancer mortality and survival groups. In addition, multivariate analysis showed that age, gender, extensive ETE, and lymph node metastasis exhibited a statistically significant difference between PTC with ETE and thyroid cancer mortality (Table 2).

**Table 2:** Univariate and multivariate analysis of clinical features of papillary thyroid cancer with extra-thyroid extension in cancer mortality or survival groups

Clinical characteristics	Cancer mortality	Survival	*P* value (univariate/multivariate*)
Total patients	28 (10.4)	241 (89.6)	
Age at diagnosis (years)	58.5 ± 14.1	45.4 ± 14.6	0.0001/0.0001
Gender (female)	13 (46.4)	191 (79.3)	0.0001/0.0036
Minimal ETE (T3)/Widely ETE (T4)	6 (21.4)/22 (78.6)	169 (70.1)/72 (29.9)	0.0001/0.0035
Mean tumor size (cm)	4.0 ± 2.1	2.9 ± 1.4	0.0003/0.3915
Post-operative serum thyroglobulin level after 1 month (ng/mL)	141.0 ± 300.3	91.3 ± 533.8	0.6419
Multifocality	8 (28.6)	57 (23.7)	0.5648
Thyroid operative method			0.2738
Total thyroidectomy	17 (60.7)	120 (49.8)	
Total thyroidectomy with lymph node dissection	11 (39.3)	121 (50.2)	
Lymph node metastases	14 (50.0)	66 (27.4)	0.0132/0.0256
Vascular invasion	2 (7.1)	8 (3.3)	0.3114
Follow-up period (years)	6.7 ± 5.4	14.1 ± 5.0	0.0001
Post-operative ^131^I accumulative dose (mCi)	324.6 ± 400.3	196.1 ± 226.3	0.0109
Diabetes mellitus	2 (7.1)	19 (7.9)	0.8900
Second primary cancer	5 (17.8)	24 (10.0)	0.2021

Number (%)

* Multivariate analysis by Cox proportional hazards regression model for survival and mortality

**Fig. 2:**
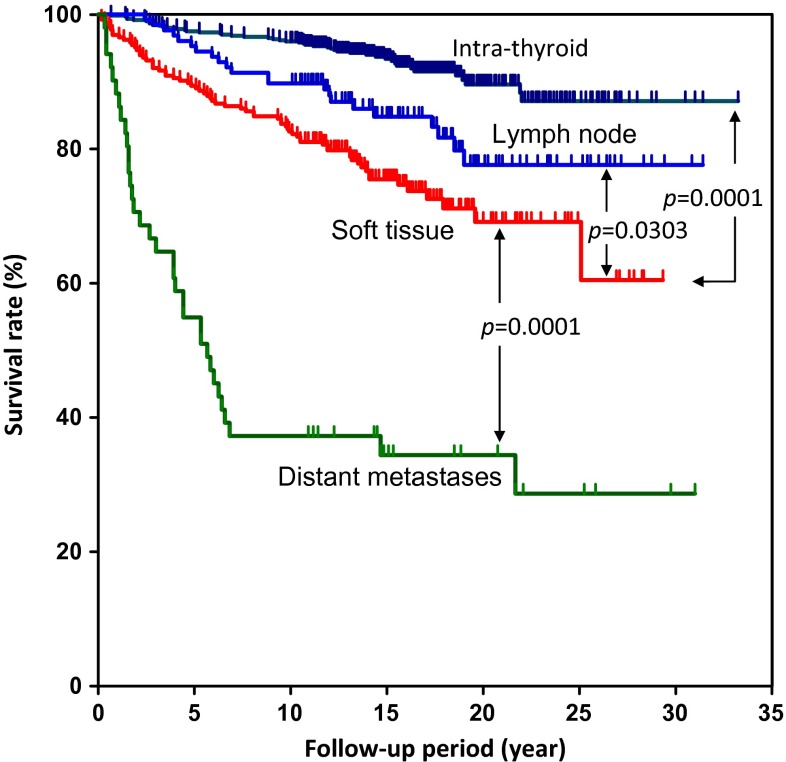
Survival rates of patients with papillary thyroid cancer in intra-thyroid, lymph-node metastases, soft tissue invasion and distant metastases groups at time of thyroidectomy

**Fig. 3:**
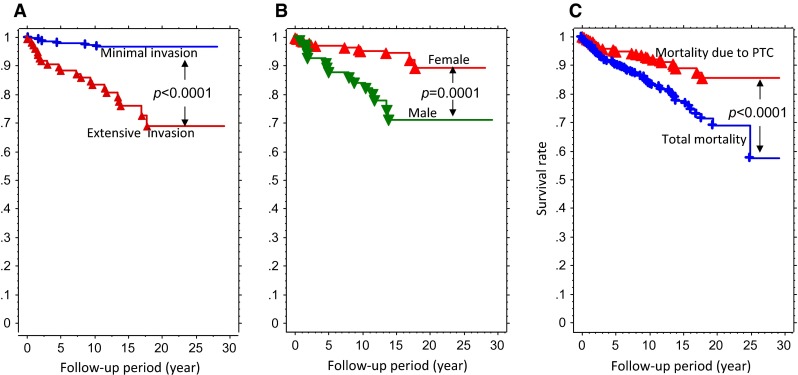
Survival rates of patients with papillary thyroid cancer with soft tissue invasion. **a** Minimal invasion and extensive invasion groups. **b** Female and male patients with papillary thyroid cancer with soft tissue invasion. **c** Total mortality and papillary thyroid cancer (PTC)-specific mortality

Figure 2 illustrated Kaplan–Meier survival curves of PTC patients in intra-thyroid, lymph-node metastases, soft tissue invasion and distant metastases groups. The survival rate of patients with soft tissue invasion was worse than intra-thyroid and lymph node metastasis; otherwise, survival rate was better than distant metastasis. The survival rates for the total ETE patient population, and minimal ETE and extensive ETE patients were 94.7, 97.7, and 87.9 % at 5 years; 92.2, 97.0, and 83.1 % at 10 years; and 95.1, 96.3, and 68.4 % at 20 years, respectively (Fig. 3a). Survival rates were significantly different between minimal ETE and extensive ETE groups (*p* < 0.0001). In addition, Kaplan–Meier survival curves illustrated that males had a worse prognosis than females (Fig. 3b). Total vs. PTC mortality of PTC with ETE showed total mortality significantly higher than thyroid cancer specific mortality (Fig. 3c).

At the end of the follow-up period, there were 16 cases (5.9 %) with locoregional invasion, and 28 (10.4 %) with distant metastases (Table 3). Thyroid cancer-related and total mortalities were higher in locoregional invasion group, although the difference was not statistically significant. The follow-up period was longer in distant metastasis group (9.5 vs. 5.9 years; *p* = 0.0322). Of the 49 patients who underwent re-operation, 29 of them initially presented with extensive ETE. The thyroid cancer-related mortality rate was higher in the patient group that underwent re-operation than in the group that did not undergo re-operation (24.5 vs. 7.3 %) (Table 4).

**Table 3:** Clinical features of papillary thyroid cancer with locoregional recurrence or distant metastasis at final follow-up status

Clinical characteristics	Locoregional	Distant metastasis	P*	Others	P**
Total patients	16 (5.9)	28 (10.4)		225 (83.6)	
Minimal ETE (T3)/widely ETE (T4)	7 (43.8)/9 (56.3)	5 (17.9)/23 (82.1)	0.0636	163 (72.4)/62 (27.6)	0.0001
Gender (female)	7 (43.8)	15 (53.6)	0.5308	182 (80.9)	0.0001
Age at diagnosis (year)	63.2 ± 10.3	52.9 ± 16.4	0.0325	44.9 ± 14.2	0.0001
Mean tumor size (cm)	4.1 ± 2.1	3.9 ± 1.7	0.7088	2.8 ± 1.3	0.0001
Post-operative serum thyroglobulin level after 1 month (ng/mL)	140.0 ± 137.1	505.5 ± 1542.7	0.3779	46.8 ± 143.8	0.0001
Multifocality	3 (18.8)	10 (35.7)	0.2354	52 (23.1)	0.2966
Thyroid operative method			0.4901		0.5495
Total thyroidectomy	8 (50.0)	17 (60.7)		112 (49.8)	
Total thyroidectomy with lymph node dissection	8 (50.0)	11 (39.3)		113 (50.2)	
TNM stage			0.0449		0.0001
Stage I	1 (6.3)	8 (28.6)		110 (48.9)	
Stage III	5 (31.3)	2 (7.1)		68 (30.2)	
Stage IV	10 (62.5)	18 (64.3)		47 (20.9)	
Follow-up period (years)	5.9 ± 4.5	9.5 ± 5.4	0.0322	14.3 ± 5.0	0.0001
Post-operative ^131^I accumulative dose (mCi)	203.6 ± 289.6	472.4 ± 375.5	0.0201	177.2 ± 208.5	0.0001
Diabetes mellitus	1 (6.3)	5 (17.9)	0.2805	15 (6.7)	0.1114
2nd primary cancer	3 (18.8)	5 (17.9)	0.9411	21 (9.3)	0.2226
Cancer mortality	11 (68.8)	15 (53.6)	0.3246	2 (0.9)	0.0001
Total mortality	14 (87.5)	17 (60.7)	0.0610	32 (14.2)	0.0001

Number (%)

* Between locoregional recurrence and distant metastasis groups

** For three groups

**Table 4:** Clinical features of papillary thyroid cancer with extra-thyroid extension underwent local neck re-operation and without re-operation groups

Clinical characteristics	Re-operation	Without re-operation	*P* value
Total patients (%)	49 (18.2)	220 (81.8)	
Mean operation times	1.5 ± 1.2	–	–
Minimal ETE (%)/Extensive ETE (%)	20 (40.8)/29 (59.2)	155 (70.5)/65(29.5)	0.0001
Gender (female)	31 (63.3)	173 (78.6)	0.0230
Age at diagnosis (years)	48.7 ± 15.8	46.4 ± 14.9	0.3194
Mean tumor size (cm)	3.7 ± 1.6	2.8 ± 1.4	0.0001
Post-operative serum thyroglobulin level after 1 month (ng/mL)	325.6 ± 1146.1	45.6 ± 142.5	0.0007
Multifocality	11 (22.4)	54 (24.5)	0.7565
Thyroid operative method			0.0260
Total thyroidectomy	32 (65.3)	105 (47.7)	
Total thyroidectomy with lymph node dissection	17 (34.7)	115 (52.3)	
TNM stage			0.0003
Stage I	20 (40.8)	99 (45.0)	
Stage III	5 (10.2)	70 (31.8)	
Stage IV	24 (49.0)	51 (23.2)	
Follow-up period (years)	12.7 ± 5.6	13.4 ± 5.5	0.4069
Post-operative ^131^I accumulative dose (mCi)	497.2 ± 380.6	153.8 ± 153.8	0.0001
Cancer mortality	12 (24.5)	16 (7.3)	0.0004
Total mortality	16 (32.7)	47 (21.4)	0.0915
Diabetes mellitus	3 (6.1)	18 (8.2)	0.6270
Radiotherapy	17 (34.7)	16 (7.3)	0.0001
Second primary cancer	7 (14.3)	22 (10.0)	0.3817
Disease free	5 (10.2)	84 (38.2)	0.0002

*ETE* extra-thyroid extension

Number (%)

## Discussion

Recent long-term follow-up studies of well-differentiated thyroid cancer and PTC revealed that the rates of all-cause mortality, recurrence, and thyroid cancer-related death increased in cases of locoregional extension of PTC [1, 9]. The clinical features of PTC patients with ETE are variable. In our study group, 18.9 % (187/2, 583 PTC cases) of PTC patients were diagnosed with soft tissue invasion. Depending on patient selection and enrolment, the incidence of ETE in PTC was variable [10–12]. Patients categorized into T3b and T4a stage groups using the TNM staging system differed in terms of clinical presentation and long-term follow-up. In our study, 65.1 % of patients with ETE exhibited minimal invasion to perithyroidal tissue. As demonstrated in previous studies, extensive invasion had a higher recurrence and cancer mortality [5, 6]. Compared with previous studies, our study had a longer follow-up period and excluding those with papillary microcarcinoma. The design of the study may include more advanced cases and administered a consistent surgical procedure.

The aggressive clinical course of PTC with ETE may result in locoregional compression in the form of airway compression, hemorrhage, superior cavernous sinus syndrome or distant metastasis [13–15]. In our study, 28 cases (10.4 %) had distant metastasis at the end of follow-up period. In previous studies, patients with locoregional invasion had higher mortality rates and a shorter follow-up period. As our study had a longer follow-up period (median: 13.5 years), a total of 16.3 % had locoregional or distant metastasis. The overall recurrence rate was higher compared to the studies of shorter follow-up period [16–18].

^131^I and external radiotherapy did not improve thyroid cancer survival in patients who had PTC with soft tissue invasion. Local control of PTC with extrathyroid invasion is very important for avoiding macroscopic residual cancer either in locoregional or in distant metastasis [19]. In a recent study, with the help of technical advances in radiotherapy planning, simultaneous integrated boost intensity modulated radiotherapy was performed in patients with locoregionally advanced papillary thyroid cancer [20]. Preliminary results showed the method proved effective in improving locoregional control in patients with locally advanced PTC.

In addition to extension of PTC, tumor size, and age, male gender was illustrated as an independent poor prognostic factor in local advanced thyroid cancer and PTC with distant metastasis [19, 21]. High prevalence of PTC is well known in female subjects. Estrogen receptor α and progesterone receptor expressions were investigated in PTC tissue and cell lines [22, 23]. BRAF (V600E) mutation was detected in 23.2 % of the tumors with a higher prevalence in larger tumors and in those with a stronger estrogen receptor α and progesterone receptor staining. These investigations suggested sex hormones play a role in PTC patient prognosis. Similar to our findings, thyroid cancer-specific survival was statistically worse in males than in females.

In our analysis, patients that belonged to PTC with extensive invasion group had 6.9 times higher thyroid cancer-specific mortality rates than those in the minimally invasive group (23.4 vs. 3.4 %). In addition, 3.5 times higher total mortality rates were observed in the group with extensive invasion when compared to that with minimal invasion (43.6 vs. 12.6 %). These results confirmed the recent study from Verburg et al. [9] reporting the impact of extensive invasion by PTC on life expectancy. Longer follow-up of PTC patients results in a higher total mortality rate than thyroid cancer-specific mortality. A second primary malignancy, DM, and other cardiovascular diseases may play important roles in the total mortality rate [21–23]. Further investigation was warranted. Similar to the previous study, patients underwent re-operation for local recurrence with higher mortality [24]. Most re-operations are performed immediately after the initial thyroidectomy and likely reflect persistent rather than recurrent disease. The limitations of this study include the change in diagnostic tools over 30 years. Furthermore, different surgeons and endocrinologists follow different therapeutic strategies. The causes of death, especially that of non-thyroid cancer, were unclear.

In conclusion, soft tissue involvement by PTC with ETE at the time of the operation is an important determining factor for long-term patient outcome. Old age, male gender, and lymph node involvement are factors that require closer follow-up and aggressive treatment. Persistent or recurrent locoregional PTC did not have better prognosis when compared to distant metastasis.
